# Report of the Physician to the Hospital for Small-Pox and Vaccination, at St. Pancras; to His Royal Highness the Duke of York, President, to the Vice-Presidents, Treasurer, and Governors of That Institution

**Published:** 1822-08

**Authors:** George Gregory

**Affiliations:** Physician to the Small-Pox and Vaccinalion Hospital, at St. Pancras. London


ART. II.—*Report of the Physician to the Hospital for Small-Pox and Vaccination, at St. Pancras; to his Royal Highness the Duke of York, President, to the Vice-Presidents, Treasurer, and Governors of that Institution.*

YOUR Physician desires to call your attention to the proceedings which have taken place within the walls of your hospital during the present year, in so far as they have fallen under his cognizance as medical attendant of the institution; and to report upon the degree of benefit, to the community at large, which your establishment during that period may claim to have effected.

Sixty-nine patients labouring under the casual small-pox have been admitted between the 1st January and 5th June, 1822. Of these, 35 have had the disease in a severe, and 34 in a mild, form: 44 have been discharged cured; 18 have died; and 7 remain under treatment. The proportion of admissions, of severe cases, and deaths, is nearly the same as that of the two preceding years; and it shows that, although small-pox has not been particularly prevalent in the metropolis, it has yet lost nothing of its malignity; and that the same necessity exists now, as formerly, for those measures of prevention and relief which it is the humane design of your institution to provide.

It is peculiarly gratifying to your physician to report, that the department of your establishment appropriated to vaccination has prospered, during the last five months, in an unexampled manner:—1524 persons have been vaccinated at the hospital during that period, being an increase of one-fourth above the numbers of the former year. It must afford great satisfaction to the governors to observe that the confidence of the public in the efficacy of vaccination would thus appear, from their books, to be on the increase: and it may be presumed that such is the general feeling in London, when it is considered that the applicants for vaccination at this hospital are not confined to its own immediate neighbourhood, but are spread over the most distant parts of the metropolis.

The regularity with which mothers attend with their children at the stated periods during the progress of vaccination, is highly exemplary; and it has the double advantage of enabling your physician to certify the correctness with which the process has been gone through, and to ensure to the public at all times an ample supply of vaccine lymph in its most perfect state.

The nature of your institution has afforded to your physician opportunities of forming a judgment on several points connected with vaccination, which attract a great share of public attention; and he is desirous of reporting to the governors to what extent he has observed small-pox to prevail after vaccination, and how far the occurrence of such cases has already influenced, or appears likely in future to influence, the practice of cow-pock inoculation.

In the present year, 22 out of the 69 who have been admitted state themselves to have undergone vaccination at some former period. Seven of them had the disease in a severe and unmitigated form, and fifteen in that mild and modified way to which the term "varioloid" is usually applied. In the larger proportion of instances, the mildness of the secondary disorder was strikingly displayed; and, within the wards of your hospital, it afforded an instructive contrast to the malignant character which the disease too frequently assumed in those who did not enjoy the protecting influence of vaccination.

In the severer cases, as no scars were apparent on the arms, it is highly probable that vaccination had totally failed, and that the patient or the parents had considered the puncture of the arm as equivalent to perfect vaccination. The most experienced practitioners, however, have frequent occasion to observe that the first insertion of the virus is ineffectual, and that the success of the process can be insured only by unremitting attention. That this may have been the source of fallacy in several of these cases, is rendered the more probable by the fact that, in every instance of small-pox subsequent to vaccination, which has occurred during the present year, the patient had been vaccinated in the country; and it is obvious how much greater is the chance that, under such circumstances, the indispensable inspection of the progress of the vehicle may have been neglected.

Your physician has had occasion to notice, that the proportion of persons vaccinated at your institution, who are known to have taken small-pox in after-life, is very small. A few cases of the kind have indeed fallen under his observation, but they were all of the mildest kind; and it is gratifying to him to perceive that these, so far from deterring from the practice of vaccination, impressed upon the parents its value, and strengthened their faith in its efficacy. Upon the whole, your physician is inclined to believe that the cases in which regular vaccination fails in imparting either a protecting or a highly-important modifying power, are so few as scarcely to merit notice, and infinitely too rare to affect, in any sensible degree, the beneficial practice of vaccination.

GEORGE GREGORY, M.D.
*Physician to the Small-Pox and Vaccination Hospital, at St. Pancras.*

*London; 6th June, 1822.*

---

